# Low-cost head-mounted camera system for medication-error detection

**DOI:** 10.1016/j.ohx.2026.e00812

**Published:** 2026-07-14

**Authors:** Solomon Nsumba, Joan Kaitesi, Dianah Namutosi, Pius Kavuma Mugagga, Joyce Nakatumba-Nabende, Peter Nabende

**Affiliations:** a College of Computing & Information Sciences, Makerere University, Kampala, Uganda; b LavingtonData, Mauritius

**Keywords:** Embedded systems, Edge computing, Additive manufacturing, Head-mounted devices, On-device inference, Low-resource settings

## Abstract

We present a low-cost, reproducible head-mounted wearable system for first-person video capture and real-time medication-workflow monitoring. The device combines a Raspberry Pi Zero W, Pi Camera Module v3, PiSugar 2 Pro battery board, active buzzer, tactile push button, and a modular 3-D-printed PLA enclosure with elastic head straps. Weighing approximately 145 g and costing about USD 100 for the core wearable components (USD 168.78 for the complete development and configuration bill of materials), the system is designed for offline operation, local storage, and low-power embedded processing. The enclosure was modeled in DesignSpark Mechanical and fabricated as modular panels with integrated mounting, ventilation, and access features. The software stack runs on Raspberry Pi OS Lite with Python/OpenCV scripts for autonomous capture, local event flagging, and audible feedback. Pilot deployment confirmed stable operation, up to 7 h of battery-powered use, and practical wearability in clinical settings. This work provides a reproducible open-hardware platform for wearable monitoring in low-resource operating room environments.

## Graphical abstract

**Figure:**
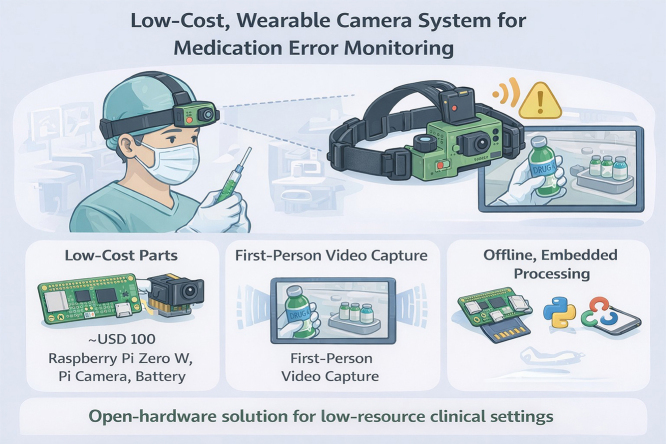

## Specifications table

**Table:** 

Hardware name	Low-Cost Head-Mounted Camera System for Medication-Error Detection
Subject area(s)	Biomedical engineering; Health informatics; Embedded systems
Hardware type	Wearable, camera-equipped monitoring device for clinical settings
Closest commercial analog	Action body cameras (e.g. GoPro) combined with barcode/AI medication-verification systems
Open-source license	CERN-OHL-S v2 (hardware) ; MIT (software)
Estimated unit cost	∼ USD 100 (Pi Zero W, camera module v3, PiSugar UPS-battery, 3-D-printed case, straps, buzzer, push-button)
Source-file repository	https://doi.org/10.5281/zenodo.19144166

## 1 Hardware in context

Preventable medication-preparation errors are a major source of peri-operative harm, particularly in low- and middle-income settings such as Uganda, where understaffing, paper-based workflows, and intermittent power often impede strict protocol adherence and safe drug administration [1], [2]. In high-income hospitals, technologies such as barcode medication administration (BCMA) systems, automated dispensing cabinets, and other closed-loop platforms reduce errors, but they come with high capital and operational costs, require robust infrastructure, and often rely on proprietary software placing them beyond the reach of most Ugandan operating theaters [3]. Even when implemented, compliance with these systems can vary, and continuous infrastructure support is necessary to maintain their effectiveness [3], [4].

In response to these challenges, we developed a standalone, head-mounted wearable camera platform designed to support future medication-error detection workflows through first-person video capture, on-device event flagging, and embedded monitoring capabilities. The primary contribution of this work is the design, fabrication, deployment, and open dissemination of a low-cost wearable hardware platform suitable for low-resource clinical environments. The system is built entirely from off-the-shelf components consisting of the Raspberry Pi Zero W, Pi Camera Module v3, PiSugar UPS battery, active buzzer, and tactile mute button which has an estimated core wearable hardware cost of approximately USD 100, weighs 145 g, and operates independently of mains power or network connectivity. A Python/OpenCV routine running on the device analyses first-person video and triggers an audible alert whenever a preparation step deviates from protocol. All CAD models, firmware, and build scripts are released under open licenses (CERN-OHL-S for hardware, MIT for software) to allow hospitals and research labs to replicate and adapt the system [5], [6].

A pilot deployment in eight surgical sessions at CoRSU Rehabilitation Hospital captured more than 20 h of footage; clinicians reported high comfort and no workflow disruption, confirming the practicality of the form factor [7]. By pairing commodity embedded hardware with open licensing, the system offers a scalable, context-appropriate pathway toward improving medication safety in resource-constrained operating rooms and aligns with wider efforts in surgical safety and digital-health equity [8].

Table 1 (System Positioning Matrix) compares system types on cost, power requirements, infrastructure, and capability for real-time versus offline use:

This positioning highlights how pairing commodity-embedded hardware with open licensing provides a cost-effective, low-infrastructure pathway to improving medication safety in resource-constrained operating rooms. It delivers real-time error detection without the expense, infrastructure dependence, or proprietary restrictions of traditional systems. Recent studies of wearable camera systems using deep learning have demonstrated high real-world sensitivity and specificity (>99%) for detecting medication errors, underscoring the feasibility and clinical promise of wearable vision approaches [8].

**Table 1:** System positioning matrix: Comparison of medication safety systems.

System type	Cost (USD)	Power	Infrastructure	Real-time	Offline
Barcode medication admin systems (BCMA)	$20,000–$200,000+ per facility	High (server + workstations)	Required (IT network, pharmacy system)	Yes (scan feedback)	No

Body-worn video systems (professional)	∼$200–$700 per unit (device only) + backend costs	Medium (battery packs 6–12 h)	No core infrastructure required, optional cloud/storage	No (capture only)	Yes (video review)

LCH-MCMED system (*ours*)	∼ŨSD 100 (core wearable components)	Low (∼0.9–1 W board power)	None (stand-alone)	Yes (real-time on-device)	Yes (local video/log)

## 2 Hardware description

The proposed device is a lightweight (**145 g**), head-mounted wearable camera platform designed to support AI-assisted medication workflow monitoring in operating-room environments. The system integrates commercially available embedded hardware with a custom 3D-printed enclosure to provide an affordable, reproducible, and portable platform for first-person video acquisition and on-device computer vision. The wearable operates entirely on battery power and performs local image acquisition, storage, and inference without requiring external computing infrastructure or network connectivity during deployment. The deployable hardware costs approximately **USD 100** per unit, making the platform suitable for low-resource healthcare settings.

### Key components

**Fig. 1:**
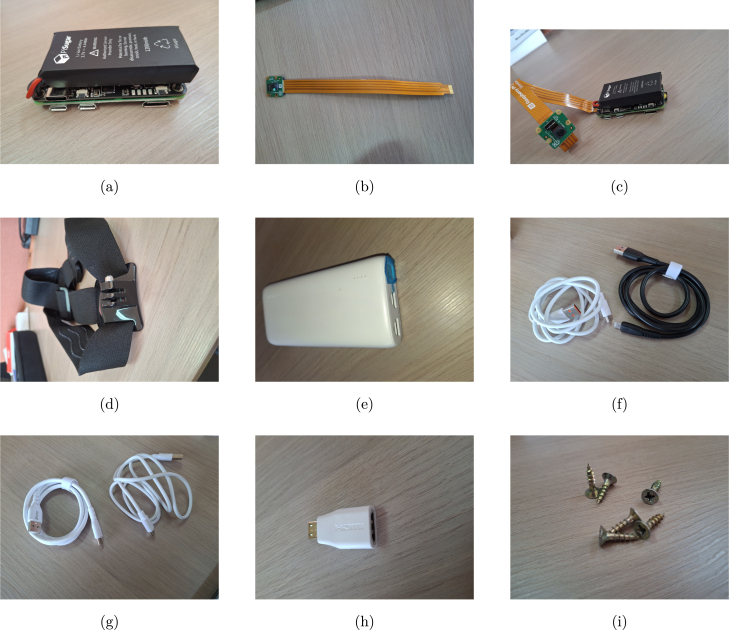
Principal hardware components used to assemble the wearable platform: (a) Raspberry Pi Zero W; (b) Raspberry Pi Camera Module v3; (c) PiSugar 2 Pro battery management module; (d) adjustable elastic head strap; (e) external USB power bank; (f) Micro-USB charging cables; (g) USB Type-C charging cables; (h) HDMI and Micro-USB OTG adapters; and (i) nylon screws and plastic standoffs.

The wearable platform is assembled entirely from commercially available embedded hardware and custom-fabricated mechanical components selected for their low cost, availability, low power consumption, and ease of replication. The principal hardware consists of a Raspberry Pi Zero W single-board computer, a Raspberry Pi Camera Module v3, a PiSugar 2 Pro battery management module, an active buzzer, a tactile push button, a custom 3D-printed PLA enclosure, adjustable elastic head straps, and supporting accessories including a microSD card, CSI ribbon cable, and external power bank. Fig. 1 illustrates the principal hardware components used to assemble the wearable platform, while the complete bill of materials, suppliers, and estimated costs are presented in Section 4.

• **Embedded computing platform:** Raspberry Pi Zero W providing local image acquisition, storage, and execution of the embedded computer vision pipeline.
• **Imaging subsystem:** Raspberry Pi Camera Module v3 with autofocus capability for first-person capture of medication preparation activities.
• **Power subsystem:** PiSugar 2 Pro battery management module together with an optional external USB power bank for extended operating time.
• **User interface:** Active buzzer and tactile push button providing audible feedback and user acknowledgement during operation.
• **Mechanical subsystem:** Modular 3D-printed PLA enclosure with passive ventilation and adjustable elastic head straps for comfortable hands-free operation.

### Enclosure and ergonomics

The enclosure clicks together with nylon screws and alignment posts, allowing fast access for maintenance or part replacement. Ventilation slits on both sides prevent thermal throttling, while integrated loops guide the elastic straps so the load is evenly distributed across the brow. The camera aperture is flush with the front panel, giving an unobstructed, sterile-field view of syringes and vials.

### Power and data flow

On boot, a Python-based acquisition and inference pipeline autostarts via /etc/rc.local, enabling fully autonomous operation without user intervention. The system records .mp4 video streams to the onboard micro-SD card while simultaneously performing on-device image analysis. During prototype deployment, a lightweight YOLOv11n object detection model integrated with OpenCV routines was used to identify medication-related objects such as syringes, vials, and labels within the field of view. Detection outputs were subsequently used to support event flagging and audible alerts when predefined mismatch conditions were identified. Upon detection of a mismatch, an audible alert is triggered via the buzzer to provide immediate feedback to the clinician.

The objective of this deployment was to demonstrate the feasibility of integrating computer vision functionality into a low-cost wearable platform operating entirely on embedded hardware. The deployed YOLOv11n model successfully demonstrated on-device inference and real-time event flagging during pilot deployment. However, the present study was not designed to comprehensively evaluate detection performance metrics such as sensitivity, specificity, precision, recall, false-positive rates, or alarm fatigue under clinical conditions. Instead, validation focused on hardware operation, usability, deployment feasibility, and the practical integration of the wearable system into a real clinical environment. Comprehensive algorithm evaluation using annotated datasets and clinically representative medication-error scenarios remains part of ongoing and future work.

A tri-color LED provides intuitive system-state feedback, indicating *stand-by*, *active recording*, and *low-battery* conditions. This form of real-time, edge-based processing aligns with emerging trends in embedded clinical monitoring systems, where immediate feedback has been shown to improve adherence to safety protocols and reduce error rates [4], [9].

**Fig. 2:**
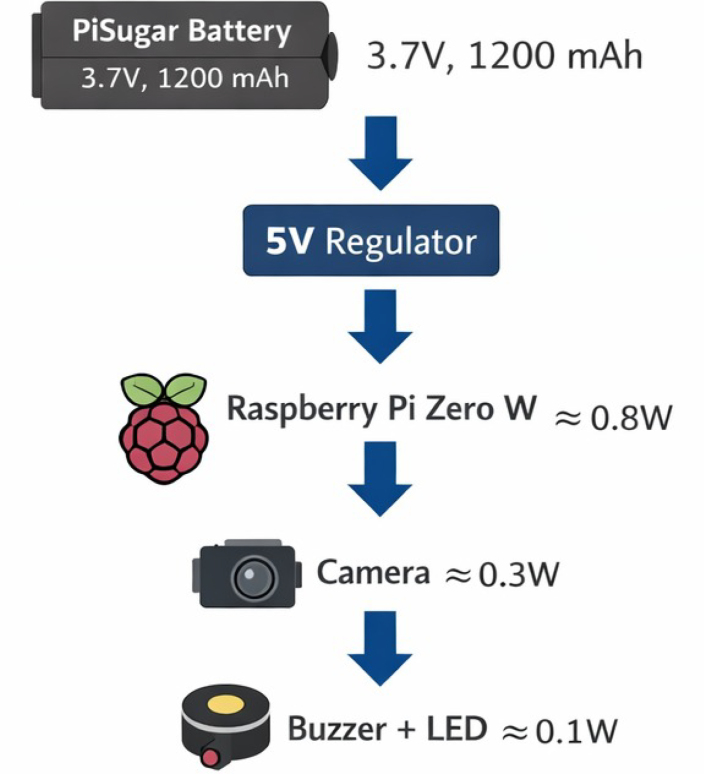
Power consumption of system components under operational states (idle and image capture), illustrating current draw variations of the Raspberry Pi Zero, PiSugar module, Raspberry Pi Camera, and buzzer.

To maintain flexibility across deployment environments, the system has been designed to support optional wireless data synchronization in future deployments. However, during the pilot evaluation reported in this study, all processing and storage were performed locally on the device, and no video data were transmitted to external servers. Recorded footage was retained on the onboard microSD card for subsequent review and analysis. Prior to archival or secondary analysis, future deployments may incorporate additional privacy-preserving mechanisms such as automated face anonymization, controlled-access storage, and encryption to further strengthen patient confidentiality. In the absence of network connectivity, all data are retained locally, ensuring uninterrupted operation in low-resource settings. This design directly addresses well-documented barriers to digital health system adoption in low- and middle-income countries, including unreliable infrastructure and limited connectivity [2]. By prioritizing local processing and storage, the system preserves data sovereignty while maintaining operational independence (see Fig. 2).

### Advantages over commercial solutions

Unlike conventional medication safety systems, which often rely on centralized infrastructure and high-cost proprietary platforms, the proposed device adopts a fully open and modular architecture. All hardware designs, firmware, and software components are released under permissive open-source licenses (CERN-OHL-S for hardware and MIT for software), enabling reproducibility and adaptation across diverse clinical contexts. Open hardware approaches have been shown to accelerate innovation and reduce barriers to adoption, particularly in resource-constrained environments [6], [7].

From a cost perspective, the system represents a significant reduction compared to existing commercial solutions such as barcode medication administration systems and professional body-worn cameras. While such systems can cost tens of thousands of dollars and require extensive backend infrastructure, the proposed device achieves comparable functionality at a fraction of the cost. The complete bill of materials, including development, charging, configuration, and debugging accessories, totals USD 168.78, while the core wearable hardware required for deployment is approximately USD 100. This cost reduction is primarily enabled by the use of commodity embedded platforms and widely available components, a strategy increasingly explored in low-cost medical device development [7].

In addition to affordability, the system is designed to operate with minimal infrastructure requirements. All processing is performed locally on the embedded device, eliminating dependence on cloud services or continuous network connectivity. This approach is particularly advantageous in settings where power and connectivity are intermittent, conditions that have been identified as major barriers to the deployment of digital health technologies in LMICs [2]. Furthermore, the modular enclosure design allows for future extensions, such as the integration of inertial measurement units (IMUs), additional camera modules, or edge AI accelerators, supporting ongoing research and system evolution.

### Representative use-cases

The system supports a range of clinical and research applications, particularly in environments where traditional digital health infrastructure is unavailable. In hospital settings lacking electronic health records or barcode-based verification systems, the device can facilitate prospective studies on medication safety by capturing and analyzing real-time preparation workflows. Such observational approaches have been widely used to quantify and understand medication errors in clinical environments [9], [10].

Beyond clinical deployment, the device also provides a low-cost alternative to professional body-worn camera systems for surgical training and simulation. First-person video capture enables detailed review of procedural workflows, supporting both education and quality improvement initiatives. Additionally, the platform can serve as a testbed for edge-AI research, including gesture recognition and workflow analysis, building on prior work in wearable health monitoring systems using embedded platforms [5].

In mobile clinics and field hospitals, where documentation tools are often limited, the system offers a portable solution for recording and reviewing clinical activities. It can also be used as a teaching aid for students and trainees, helping reinforce correct medication preparation practices and adherence to sterile technique. By combining affordability, portability, and real-time feedback, the device provides a practical and scalable approach to improving medication safety in resource-constrained settings, aligning with broader global health priorities for safe and high-quality care delivery [8].

### Potential applications and adaptability

Beyond medication-workflow monitoring, the proposed wearable platform provides a flexible open-source foundation that can be adapted for a wide range of embedded sensing and computer vision applications. Potential uses include:

• **Wearable computer vision research:** The platform can be used to acquire first-person image and video datasets for object detection, activity recognition, workflow analysis, and egocentric vision research.
• **Clinical workflow monitoring:** Researchers can adapt the hardware to investigate surgical workflows, hand hygiene compliance, instrument usage, procedural documentation, and other patient-safety applications.
• **Edge AI and embedded systems evaluation:** The Raspberry Pi Zero W-based architecture provides an inexpensive platform for benchmarking lightweight deep-learning models, edge-computing algorithms, and hardware accelerators under realistic operating conditions.
• **Education and laboratory teaching:** Because all hardware designs, software, and assembly instructions are openly available, the system can serve as a low-cost educational platform for teaching embedded systems, computer vision, medical device prototyping, and additive manufacturing.
• **Adaptation to other application domains:** The modular enclosure and open hardware architecture allow straightforward integration of additional sensors such as inertial measurement units (IMUs), environmental sensors, depth cameras, or alternative imaging modules, enabling applications in industrial inspection, laboratory automation, wildlife monitoring, agriculture, and human activity recognition.

## 3 Design files summary

This section summarizes the design files required to reproduce the wearable system. The files include mechanical CAD models of the enclosure, software scripts for device operation, and associated documentation. All design files are openly available through the Zenodo repository listed in the Specifications Table to facilitate reproduction, modification, and reuse.

Table 2 provides an overview of the design files created for the development of the head-mounted wearable system. These files include mechanical models of the enclosure as well as software scripts for system operation. All design files will be made openly available in an online repository upon publication.

**device_topcover.stl:** The top panel of the enclosure, which includes a circular opening to accommodate the Pi Camera lens for unobstructed forward-facing video capture.

**Table 2:** Summary of design files used in hardware fabrication and system control.

Design filename	File type	License	Location
device_topcover.stl	STL (3D model)	CERN-OHL	/3DModels
device_rightpanel.stl	STL (3D model)	CERN-OHL	/3DModels
device_leftpanel.stl	STL (3D model)	CERN-OHL	/3DModels
device_bottompanel.stl	STL (3D model)	CERN-OHL	/3DModels
camera_timelapse.sh	Shell script	MIT	/scripts
mismatch_detector.py	Python script	MIT	/scripts

**device_rightpanel.stl:** A fully enclosed side panel with no external cutouts, providing structural integrity to the right side of the housing.

**device_leftpanel.stl:** This panel mirrors the right side in shape but includes multiple functional cutouts: a port for micro-USB (power input from PiSugar or power bank), a cutout for the PiSugar’s physical power button, an accessible opening for the tactile push button, aligned with the GPIO pin it is wired to, a Type-B USB slot, and a mini HDMI opening for display setup or debugging.

**device_bottompanel.stl:** Serves as the base of the enclosure where both the Raspberry Pi Zero W and the PiSugar 2 Pro are mounted. The PiSugar board stacks directly on top of the Pi’s GPIO header. Additional mounting posts secure the stack in place. The base also features integrated mounting buckles for fastening elastic head straps, which are secured using screws for reliable forehead attachment.

**camera_timelapse.sh:** A shell script configured in cron to run automatically on system boot. This script launches the real-time inference model

**mismatch_detector.py:** Responsible for detecting label mismatches and controlling the buzzer/button response loop.

All files are licensed under open-source terms (CERN-OHL for hardware and MIT for software) to ensure reusability and transparency. STL files were generated using DesignSpark Mechanical and verified via PrusaSlicer before 3D printing. Scripts are compatible with Raspbian Lite running on the Raspberry Pi Zero W.Fig. 33D design views of the wearable system casing modeled using DesignSpark Mechanical and fabricated using a Prusa MK3 3D printer: (a) top cover; (b) bottom cover; (c) left side panel with functional cut-outs for ports and controls; and (d) right side panel providing structural enclosure.Fig. 3

**(a):**
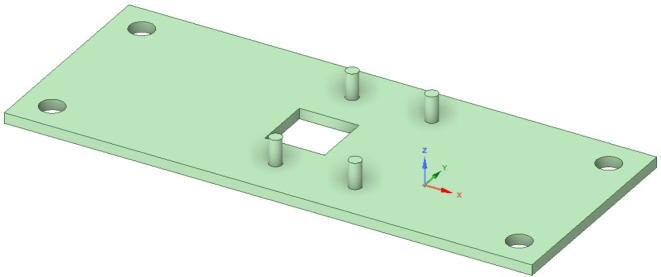
.

**(b):**
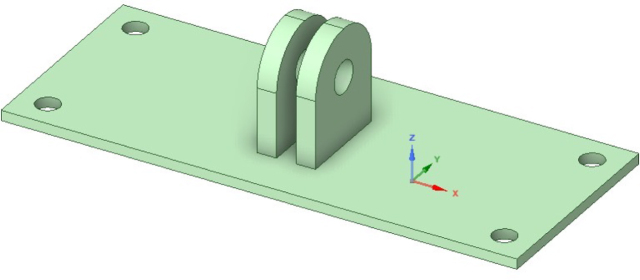
.

**(c):**
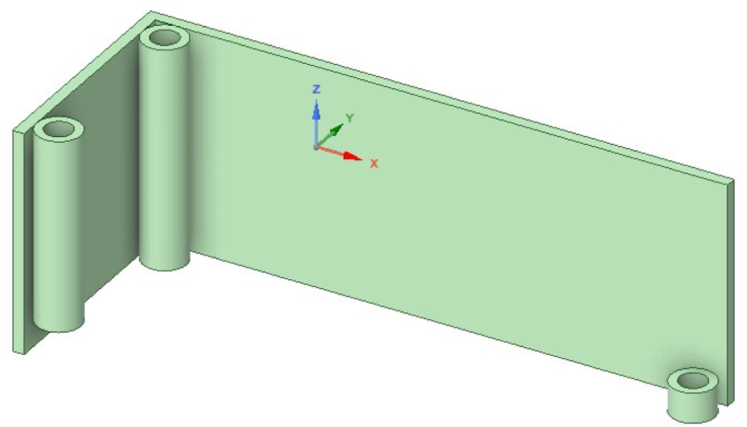
.

**(d):**
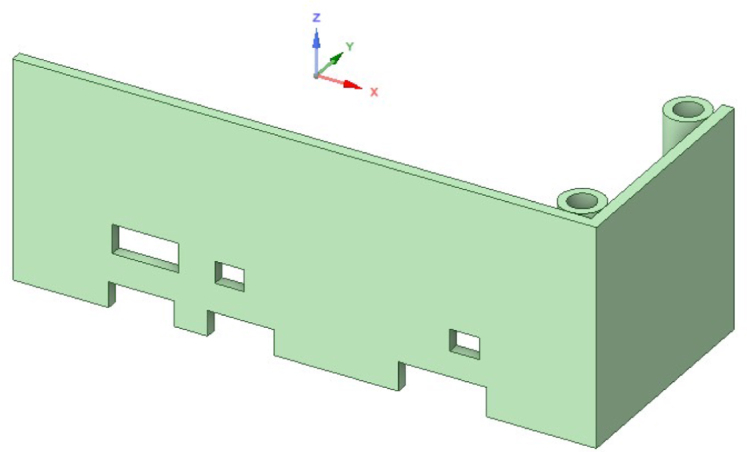
.

For full transparency and to support replication, all files used to fabricate or configure this device (STL files for the enclosure, camera mounting brackets, and Python scripts for operation) are available in the design file summary and were published in an open-source repository. Components such as the Pi Camera Module, PiSugar board, and Raspberry Pi Zero W, tactile push button, active buzzer are standard, globally available modules commonly used in embedded and IoT development. Materials were selected for their availability, affordability, and suitability for low-resource clinical environments.

DesignSpark Mechanical was used to model the enclosure with attention to compactness, heat dissipation, and weight distribution. The prototype was printed using PLA material, chosen for its lightweight, non-conductive properties. Iterative prototyping was guided by real-world feedback from initial fittings, resulting in improvements to strap placement, casing curvature, and camera module orientation.

**Fig. 4:**
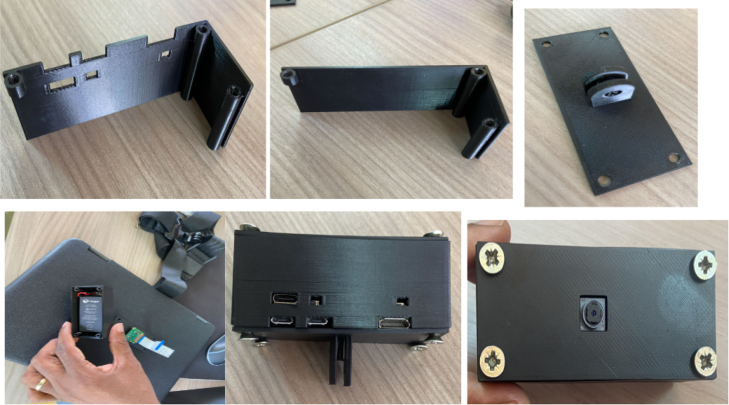
Fabricated enclosure components after 3D printing, corresponding to the original CAD designs.

The final design was exported in STL format and processed using PrusaSlicer, with profiles optimized for a Prusa i3 MK3 3D printer. The enclosure was printed using PLA filament, selected for its lightweight and ease of prototyping. All design files, including source CAD models, STL exports, and slicing settings, are provided in the supplementary materials for reproducibility and further customization by researchers or clinicians (see Fig. 4).

## 4 Bills of materials summary

The bill of materials required to reproduce the proposed wearable platform is summarized in Table 3. The listed components include both the core wearable hardware and the accessories required for system configuration, charging, debugging, and deployment.

It is important to distinguish between the cost of the deployable wearable unit and the complete development bill of materials. The complete bill of materials totals USD 168.78 and includes development and configuration accessories such as a keyboard, mouse, HDMI adapters, charging cables, wall chargers, and debugging peripherals. These accessories are required during system assembly and software configuration but are not necessary during routine deployment.

The deployable wearable platform itself consists of the Raspberry Pi Zero W, Pi Camera Module v3, PiSugar 2 Pro battery module, microSD card, CSI ribbon cable, active buzzer, tactile push button, 3D-printed enclosure, mounting hardware, and adjustable head straps. These components have an estimated hardware cost of approximately USD 100 per unit, making the platform substantially less expensive than commercial medication safety systems while remaining fully reproducible using commodity components.

The complete bill of materials is provided below to facilitate hardware replication, cost estimation, sourcing of components, and future modification by other researchers.

**Table 3:** Complete Bill of Materials (BoM) for the wearable platform. The table includes both the deployable hardware components and the additional accessories required for system configuration, charging, and software development.

Ref.	Component	Qty	Unit cost (USD)	Total (USD)	Supplier	Material
Core deployable wearable hardware						

MCU1	Raspberry Pi Zero W	2	10.00	20.00	Raspberry Pi Foundation	Semiconductor
CAM1	Pi Camera Module v3	2	25.00	50.00	Raspberry Pi Foundation	Semiconductor
BAT1	PiSugar 2 Pro battery board	2	14.99	29.98	PiSugar Official Store	Electronic/Composite
BAT2	USB Power Bank (12 000 mAh)	1	12.00	12.00	Local electronics supplier	Electronic/Composite
ENC1	3D-printed PLA enclosure	2	2.00	4.00	In-house 3D printing lab	PLA polymer
STR1	Adjustable elastic head straps	2	0.50	1.00	Local textile supplier	Fabric/Polymer
STO1	32 GB microSD card	2	6.00	12.00	Electronics retailer	Semiconductor
CAB1	CSI ribbon cable	2	1.50	3.00	Raspberry Pi accessories	Polymer/Copper
MNT1	Nylon screws and plastic standoffs	8	0.10	0.80	Hardware supplier	Nylon polymer
BUZ1	Active buzzer module	1	0.80	0.80	Electronics supplier	Electronic
BTN1	GPIO tactile push button	1	0.50	0.50	Electronics supplier	Electronic/Plastic
WIR1	Jumper wires (10 pcs)	1 set	1.00	1.00	Electronics accessories kit	Copper/Plastic

Development, configuration and charging accessories						

CAB2	USB Type-C charging cable	3	1.50	4.50	Local electronics supplier	Polymer/Copper
CAB3	USB Type-B charging cable	3	1.50	4.50	Local electronics supplier	Polymer/Copper
CHG1	USB wall charger	3	3.00	9.00	Local retailer/Amazon	Electronic
OTG1	Micro-USB OTG adapter	1	2.00	2.00	Electronics supplier	Electronic/Plastic
OTG2	Mini-HDMI adapter	1	3.00	3.00	Electronics supplier	Electronic/Plastic
PER1	USB keyboard	1	8.00	8.00	Office electronics supplier	Plastic/Electronic
PER2	USB mouse	1	5.00	5.00	Office electronics supplier	Plastic/Electronic

–	**Total bill of materials**			**USD 168.78**		

## 5 Build instructions

**Fig. 5:**
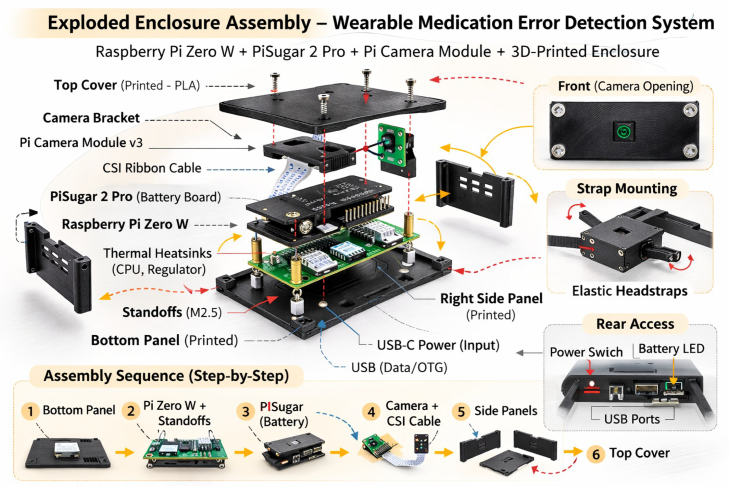
Assembly procedure of the proposed device showing the sequential integration of the Raspberry Pi Zero, PiSugar power module, Raspberry Pi Camera, buzzer unit, and custom enclosure components. The figure illustrates mechanical mounting and electrical interconnections required to replicate the system.

**Fig. 6:**
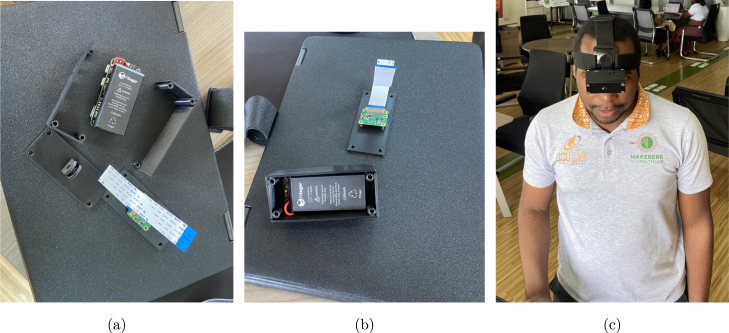
Stages of assembling the wearable system: (a) individual 3D-printed enclosure components; (b) assembled enclosure showing internal placement of electronic components; and (c) fully assembled wearable device ready for deployment.

Iterative prototyping was conducted over multiple 3D-printing cycles using a Prusa fused-filament fabrication (FFF) printer to refine dimensional tolerances, port alignment, and internal clearances. Several enclosure revisions were evaluated to achieve reliable mechanical fit and consistent assembly. Final geometries were validated across multiple print batches to confirm robustness and reproducibility under typical desktop-manufacturing variability (see Fig. 5, Fig. 6).

The construction process for the wearable medication error detection system involves mechanical fabrication, electronic assembly, and software setup. The following steps outline the full build process:

1. **3D Print Enclosure Components:** All structural parts were designed in DesignSpark Mechanical and exported as STL files, including the top cover, bottom panel, left and right side panels, and camera bracket (see Fig. 3). Parts were printed in PLA on a Prusa i3 MK3 printer with a 0.2 mm layer height and 20% infill. PLA was chosen for its low warping, ease of printing, dimensional fidelity, and skin-contact biocompatibility. The 20% infill provides sufficient rigidity while maintaining low weight for head-mounted comfort. After printing, supports were removed and edges deburred to ensure smooth fits, proper airflow, and consistent assembly.
2. **Mount Raspberry Pi Zero W:** The Pi Zero W is secured on the bottom panel using plastic standoffs and M2.5 nylon screws. Passive heat sinks are applied to the CPU and power regulators with thermal adhesive. Placement ensures unimpeded access to the CSI port, microSD slot, and GPIO headers.
3. **Attach PiSugar 2 Pro Battery Module:** The PiSugar 2 Pro plugs directly into the GPIO header and is secured using thermal tape or mounting posts. Ensure the battery is fully charged via USB-C, with the power switch and LED indicators accessible for monitoring.
4. **Install and Align Pi Camera Module:** The camera module (v2 or v3) connects to the CSI port with a ribbon cable and mounts to the camera bracket. The lens must align with the front slot for an unobstructed first-person view. Optional foam or light adhesive can stabilize the module and reduce vibrations.

### Electronic assembly and wiring

5. **Wire Optional LEDs and Buzzer:** Connect visual indicators (tri-color LED) and buzzer to GPIO pins according to the schematic. These provide feedback for *stand-by*, *recording*, and *mismatch detected* states.
6. **Head-Mount Integration:** Thread adjustable elastic straps through side loops of the enclosure. Straps must be tight enough to prevent motion but comfortable for extended wear, including with surgical caps.

## 6 Operation instructions

This section provides reproducible instructions for configuring, operating, and maintaining the proposed head-mounted wearable system. The procedures include one-time software setup, autonomous operation, remote file management, battery monitoring, and recommended practices for reliable deployment in clinical environments.

### One-time configuration

1. Download the latest Raspberry Pi OS Lite image and flash it to a 32 GB microSD card using Raspberry Pi Imager.
2. Enable SSH by creating an empty file named

**Figure:**
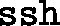

in the boot partition.
3. Configure Wi-Fi by creating a wpa_supplicant.conf file containing the local network credentials.
4. Insert the microSD card into the Raspberry Pi Zero W, power the device, and connect using

**Figure:**
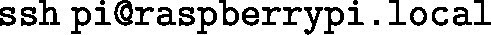
5. Enable the camera and update packages

**Figure:**
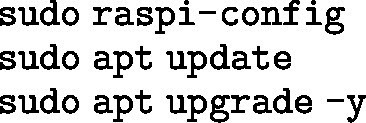
6. Install the required dependencies

**Figure:**
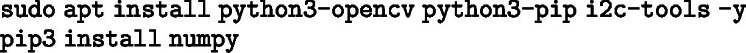
7. Copy the project scripts and trained model to

**Figure:**
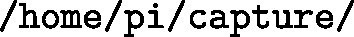

The main script initializes the camera, loads the object detection model, performs local inference, triggers audible alerts for detected mismatch events, and stores timestamped videos locally.
8. Configure automatic startup using

**Figure:**
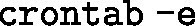

and add

**Figure:**
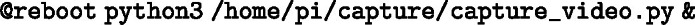

so that the wearable starts automatically after boot.
9. Verify execution permissions

**Figure:**
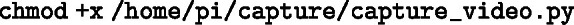

and test the system before deployment.

### Operating the device

1. Ensure that the PiSugar battery is fully charged.
2. Press and hold the PiSugar power button for approximately three seconds to boot the Raspberry Pi Zero W.
3. Secure the wearable using the adjustable elastic straps and align the camera centrally on the forehead to obtain a stable first-person view.
4. Following boot, the acquisition script starts automatically and begins continuous video capture, local object detection, and event flagging without user interaction.
5. LED indicators provide visual feedback on recording status and battery condition, while the active buzzer alerts the user when a predefined medication mismatch event is detected.
6. Videos and event logs are stored locally on the microSD card using timestamped filenames.
7. To safely power down the device, execute

**Figure:**
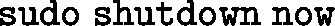

or hold the PiSugar power button for approximately three seconds until shutdown completes.

### Remote access and file management

Remote administration can be performed through SSH:

**Figure:**
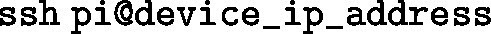

Videos can be copied from the device using

**Figure:**
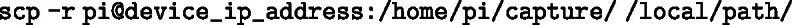

Updated scripts or models can be uploaded using

**Figure:**
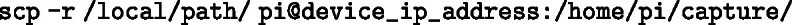

Old recordings may be removed using

**Figure:**
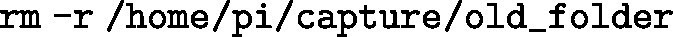

to recover storage space.

### Battery monitoring

Battery status can be queried using the PiSugar I^2^C interface

**Figure:**
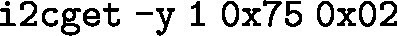

allowing operators to verify sufficient remaining battery capacity before lengthy surgical procedures.

### Best practices

• Verify camera alignment before every deployment.
• Ensure sufficient free storage on the microSD card.
• Regularly back up recorded data.
• Avoid adjusting the wearable during sterile procedures.
• Periodically inspect straps, connectors, and enclosure fasteners for wear.

### Integration into clinical workflow

The wearable is designed to integrate seamlessly into routine clinical practice. Automatic startup, passive first-person recording, and local on-device processing minimize user interaction and reduce disruption to existing workflows. Timestamped recordings facilitate retrospective auditing and quality improvement while maintaining independence from network connectivity or cloud infrastructure. The lightweight form factor enables prolonged wear during surgical procedures, making the platform suitable for deployment in resource-constrained healthcare settings.

## 7 Validation and characterization

The present study was designed primarily as a hardware prototyping and deployment study rather than a formal clinical evaluation of medication-error detection performance. Although the wearable platform incorporated a YOLOv11n object detection component for medication workflow monitoring, the primary objective of the current work was to evaluate hardware operation, deployment feasibility, wearability, usability, battery performance, and integration into routine clinical workflows. The deployed model demonstrated the feasibility of on-device inference and real-time event flagging within a low-cost wearable platform. However, extensive quantitative evaluation of detection performance metrics, including sensitivity, specificity, precision, recall, false-positive rates, and alarm fatigue, was outside the scope of the present study. Comprehensive algorithm validation using annotated datasets and clinically representative medication-error scenarios remains ongoing work and will be reported in future studies.

The system was evaluated through a pilot deployment at CoRSU Rehabilitation Hospital during eight surgical sessions involving anesthesia providers and surgical nurses. In total, the device captured over 20 h of first-person video data across peri-operative workflows. The evaluation focused on operational reliability, usability, and suitability for medication preparation monitoring, a process widely recognized as a high-risk stage for medication errors in clinical settings [4], [9].

### Deployment protocol

The wearable device was deployed in operating theatres and pre-operative preparation areas, where medication preparation and administration activities occur. Each session lasted between **15 and 45 min**, corresponding to the duration of drug preparation and early intra-operative procedures. The device was mounted using an adjustable head strap and aligned to provide a stable first-person view (FPV), enabling continuous capture of critical actions such as syringe filling, vial handling, and label inspection.

Prior to each session, the system was configured by the research team, including battery charging, camera alignment, and verification of the Python-based acquisition pipeline. The system booted autonomously and initiated recording without user interaction, supporting seamless integration into clinical workflows.

### Operational performance

The device demonstrated stable performance across all deployment sessions. Continuous video capture was maintained without significant data loss, and the system operated for up to **7 h per charge**, exceeding the duration of typical surgical lists in the study environment. The recorded video resolution and field of view were sufficient to capture fine-grained details such as medication labels and hand movements, which are essential for downstream computer vision analysis (see Fig. 7).

The system’s edge-computing architecture enabled local processing and storage, eliminating reliance on external infrastructure. This design choice is particularly important in low-resource environments, where unreliable connectivity and limited IT infrastructure have been identified as major barriers to digital health system deployment [2]. Similar trends toward edge-based clinical monitoring have been reported in recent studies on wearable and embedded healthcare systems [11].

Fig. 7Deployment and operation of the wearable system: (a) clinician wearing the head-mounted camera during medication preparation; and (b) representative first-person frame captured by the device showing medication handling during drug preparation.Fig. 7

**(a):**
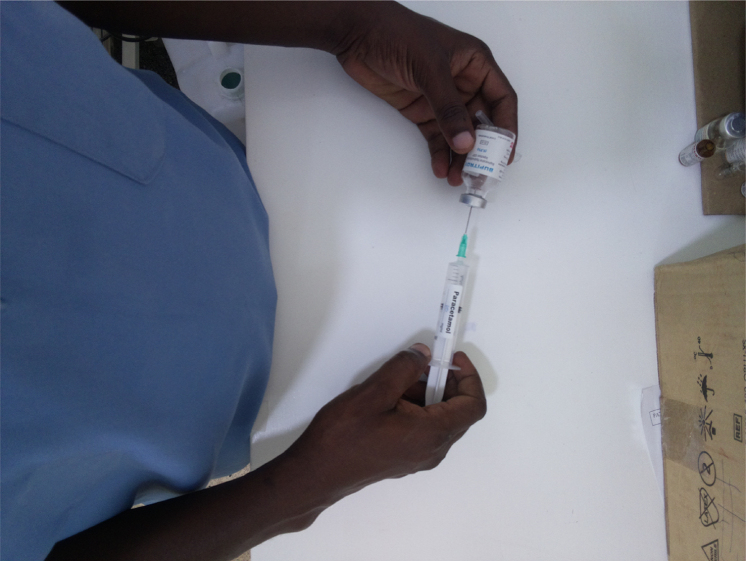
.

**(b):**
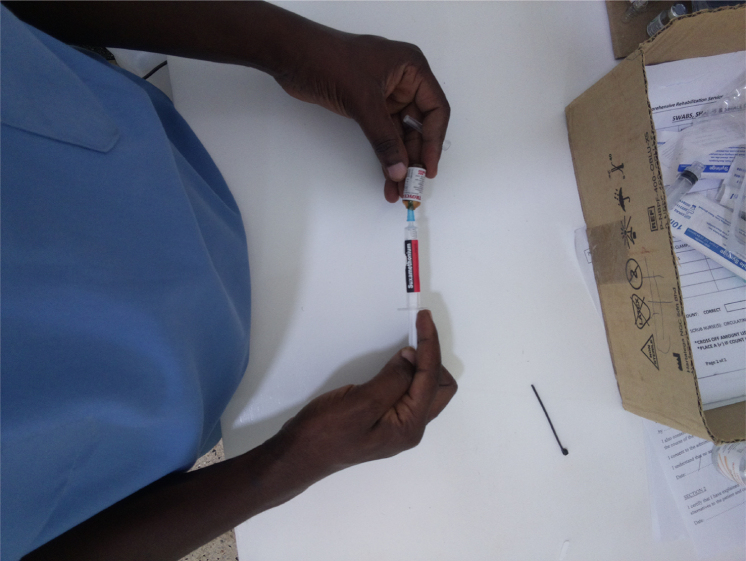
.

### Embedded detection and algorithmic evidence

Although the primary contribution of this HardwareX manuscript is the design, fabrication, and deployment of an open-hardware wearable platform, the embedded detection functionality was developed and evaluated using annotated operating-room medication-preparation datasets collected during real clinical workflows. Head-mounted video recordings were converted into annotated frames, and syringes, vials, syringe labels, and vial labels were manually annotated using CVAT. Completed annotations were reviewed by two dedicated reviewers and subsequently verified by four anesthesiologist reviewers before export in YOLO format. The resulting dataset included 7371 training images, 7337 held-out test images, and 10,658 labeled instances across 20 medication-related classes. A summary of the dataset characteristics, annotation procedures, model evaluations, and medication-error detection results is provided in Table 4.

Using this dataset, YOLOv11n and RT-DETR models were trained and evaluated for syringe and vial drug-label detection. YOLOv11n achieved a precision of 0.861, recall of 0.819, mAP50 of 0.841, and mAP50–95 of 0.453, while RT-DETR achieved a precision of 0.890, recall of 0.852, mAP50 of 0.862, and mAP50–95 of 0.480. In evaluating vial-swap detection, 253 valid draw-up events and 165 vial-swap error events were assessed, achieving 99.6% sensitivity and 98.8% specificity for vial-swap error detection. These results provide quantitative evidence supporting the feasibility of wearable-camera-based medication-error detection and complement the hardware-focused validation presented in this study.

The object-detection performance of both models is illustrated in Fig. 8. The figure presents a representative medication-preparation scene containing multiple syringes, vials, and labels in a cluttered operating-room environment. Both RT-DETR and YOLOv11n successfully identified the propofol vial and syringe; however, RT-DETR detected a greater number of relevant objects and demonstrated more precise localization in the visually complex scene. YOLOv11n also successfully identified the clinically relevant objects required for medication-workflow monitoring, although with slightly lower detection performance. This observation is consistent with the quantitative evaluation results reported in Table 4, where RT-DETR achieved marginally higher precision, recall, and mAP values than YOLOv11n. Nevertheless, YOLOv11n was designed to prioritize computational efficiency and deployment feasibility, making it more suitable for resource-constrained edge devices such as the Raspberry Pi Zero W.

**Table 4:** Summary of algorithmic evidence supporting the embedded medication-workflow monitoring pipeline.

Evaluation component	Evidence available	Relevance to current HardwareX manuscript
Ground-truth annotation	CVAT bounding-box annotation of syringes, vials, syringe labels, and vial labels; review by technical reviewers and anesthesiologists	Establishes that the detection pipeline was trained and evaluated using clinically reviewed labels
Dataset size	7371 training images; 7337 held-out test images; 10,658 labeled instances across 20 classes	Provides quantitative basis for the medication-object detection pipeline
Object detection models	YOLOv11n and RT-DETR evaluated on CoRSU operating-room data	Supports the selection of lightweight YOLO models for embedded deployment
YOLOv11n performance	Precision 0.861; recall 0.819; mAP50 0.841; mAP50–95 0.453	Demonstrates feasible detection of medication-related objects using a lightweight detector
RT-DETR performance	Precision 0.890; recall 0.852; mAP50 0.862; mAP50–95 0.480	Provides a higher-accuracy benchmark for comparison
Vial-swap detection	99.6% sensitivity and 98.8% specificity	Demonstrates that wearable-camera medication-error detection is feasible
Timing evidence	YOLO nano achieved approximately 56.3 ms processing time (∼17 FPS) during model evaluation	Supports the use of lightweight YOLO architectures for embedded deployment

The evidence summarized in Table 4 and Fig. 8 demonstrates that the medication-workflow monitoring pipeline deployed within the wearable platform was trained and evaluated using clinically reviewed operating-room datasets. To further characterize deployment on the target hardware platform, both YOLOv11n and RT-DETR were evaluated on the Raspberry Pi Zero W. The observed deployment characteristics are summarized in Table 5.Fig. 8Comparison of RT-DETR and YOLOv11n object-detection performance in an operating-room medication-preparation scene. (a) Original image showing a clinician drawing medication into a syringe from a propofol vial in a cluttered preparation environment containing multiple syringes, vials, and labels. (b) RT-DETR detection results highlighting the propofol vial and syringe using transformer-based object detection, demonstrating strong localization performance in a visually complex scene. (c) YOLOv11n detection results on the same image. Although slightly fewer objects were detected, the model successfully identified the clinically relevant syringe and vial required for medication-workflow monitoring. The comparison illustrates the trade-off between detection performance and computational efficiency that motivated the selection of YOLOv11n for deployment on the Raspberry Pi Zero W.Fig. 8

**(a):**
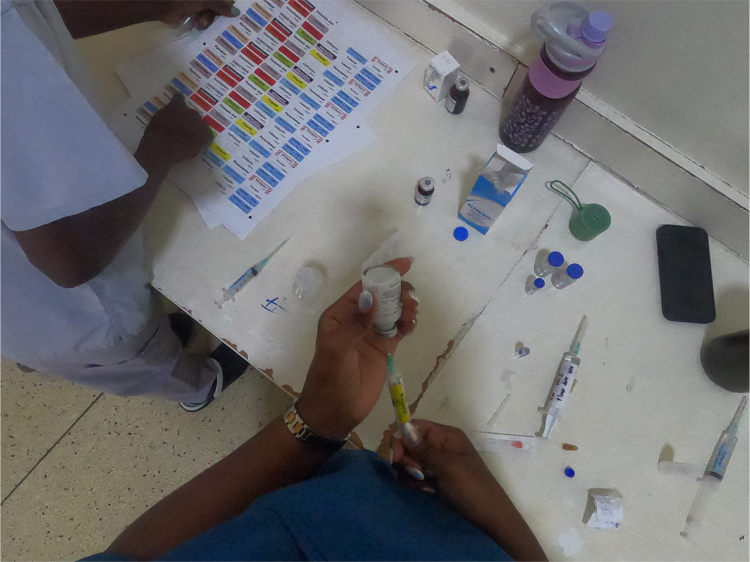
.

**(b):**
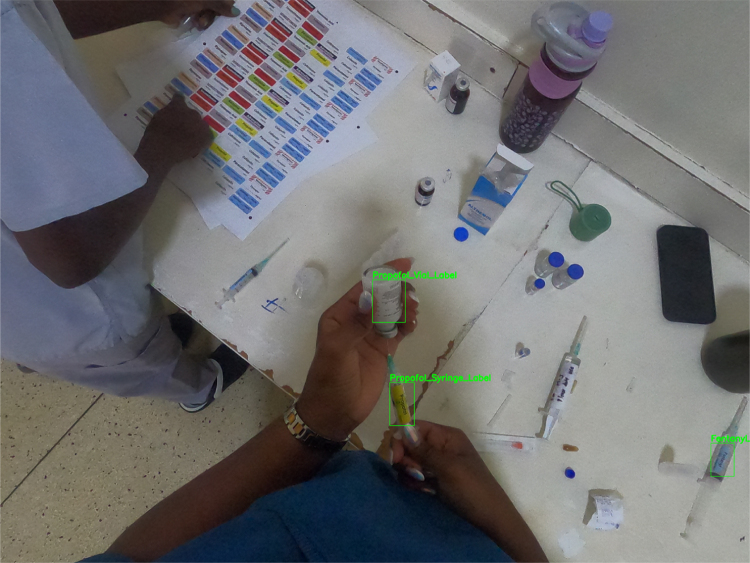
.

**(c):**
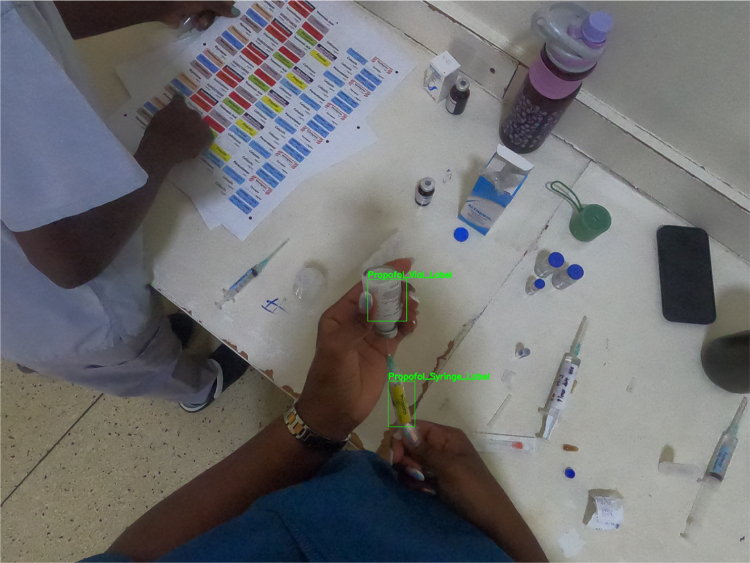
.

The deployment results summarized in Table 5 illustrate the computational challenges associated with executing modern deep-learning object detectors on highly resource-constrained embedded hardware. While both YOLOv11n and RT-DETR successfully executed entirely on the Raspberry Pi Zero W without reliance on external servers, substantial differences in inference performance were observed. YOLOv11n achieved inference latencies between approximately 12 and 26 s per frame, whereas RT-DETR required between approximately 107 and 212 s per frame under the tested conditions. Although RT-DETR achieved slightly superior detection performance during offline evaluation, its significantly higher computational cost limited its suitability for deployment on the wearable platform. Consequently, YOLOv11n was selected for prototype integration because it provided the most practical trade-off between detection performance, computational efficiency, and deployability on low-cost edge hardware.

**Table 5:** Observed deployment characteristics of YOLOv11n and RT-DETR on the Raspberry Pi Zero W.

Metric	YOLOv11n	RT-DETR
Device	Raspberry Pi Zero W	Raspberry Pi Zero W
Processor	1 GHz ARM11	1 GHz ARM11
Memory	512 MB RAM	512 MB RAM
Input resolution	480 × 800	640 × 640
Processing mode	Fully on-device	Fully on-device
Video storage	Local microSD	Local microSD
Network dependency	None	None
Observed inference latency	12.1–26.2 s/frame	107.1–212.2 s/frame
Observed frame rate	0.01–0.08 FPS	0.005–0.009 FPS
Observed CPU utilization	11.0–44.3%	11.9–17.1%
Observed RAM utilization	14.8–69.2 MB	66.4–103.2 MB
Detection output	Syringe and vial labels detected	Syringe and vial labels detected
Deployment suitability	Selected for wearable deployment	Evaluated as comparison baseline

The deployed YOLOv11n model was executed without quantization or hardware acceleration, representing a baseline implementation intended to evaluate deployment feasibility on commodity embedded hardware. Future work will investigate model quantization, pruning, and dedicated edge-AI accelerators to improve inference speed, energy efficiency, and embedded deployment performance.

The present HardwareX study focuses primarily on the design, fabrication, deployment, and embedded execution of the wearable platform rather than a prospective clinical effectiveness evaluation of medication-error prevention. Nevertheless, the results presented in this section demonstrate that clinically relevant medication objects can be detected using lightweight deep-learning models and deployed entirely on low-cost embedded hardware without reliance on external computing infrastructure. All processing and storage during deployment were performed locally on the Raspberry Pi Zero W. Future work will focus on improving inference speed, evaluating alarm fatigue, and conducting larger-scale prospective clinical studies to quantify the impact of the system on medication-error prevention and workflow safety.

### Usability and workflow integration

User feedback was collected informally from participating clinicians. The device achieved a mean comfort rating of approximately **8/10** and a perceived utility rating of **9/10**. Importantly, no participants reported significant interference with their field of view or disruption to routine clinical tasks. The lightweight design (∼145 g) and compact form factor contributed to minimal cognitive and physical burden during use.

**Fig. 9:**
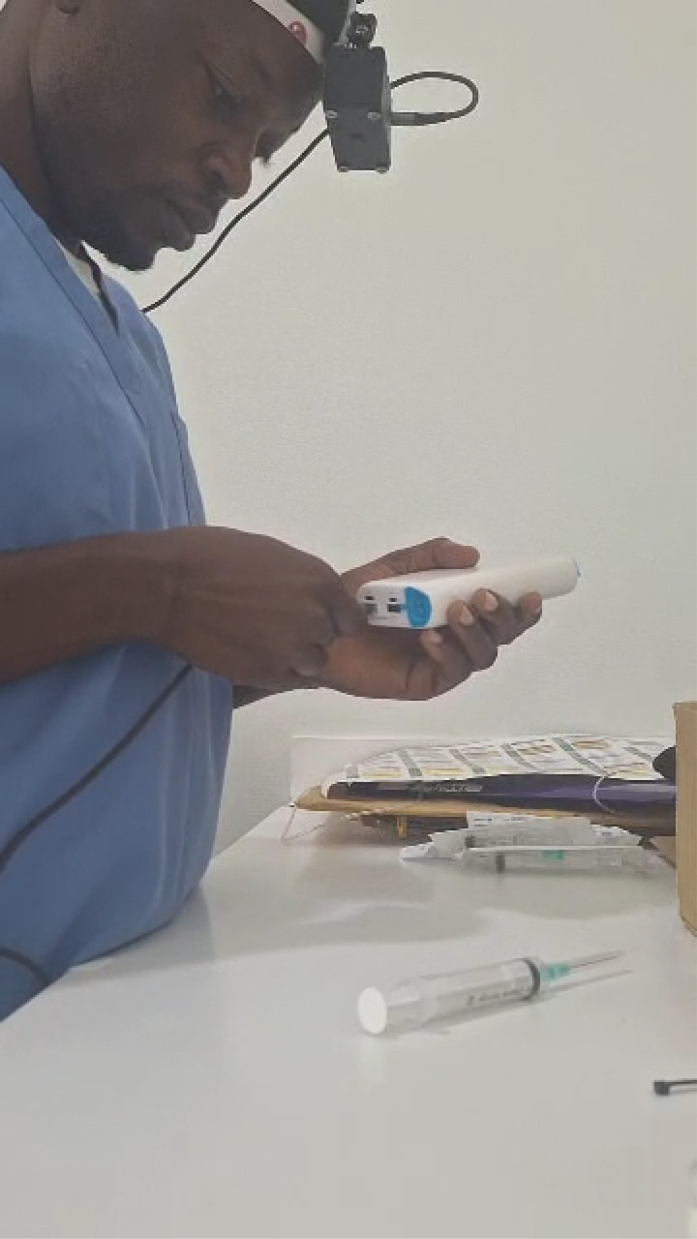
A medical provider wearing the head-mounted camera system during surgical preparation.

The ability to passively capture workflow without requiring active user input is a key advantage, as manual safety interventions such as checklists and verbal confirmations are known to be susceptible to fatigue and omission [12]. Continuous, objective recording provides an auditable record of medication preparation, supporting both retrospective analysis and future real-time decision support (see Fig. 9).

### Functional capabilities

The prototype system supports the following capabilities:

• Continuous first-person video capture of medication preparation workflows
• On-device storage and optional wireless synchronization
• Real-time event flagging using a lightweight YOLOv11n object detection pipeline integrated with OpenCV processing routines
• Autonomous operation with minimal user interaction
• Battery-powered operation suitable for low-resource settings

These features position the device as a practical tool for both observational studies and future real-time error detection systems.

### Limitations and future improvements

Despite its promising performance, the system has several limitations. First, while companion algorithmic studies provide quantitative evidence for syringe, vial, and drug-label detection, as well as vial-swap detection, the present HardwareX study was not designed as a prospective clinical effectiveness trial. The current manuscript therefore distinguishes between previously reported algorithmic validation and the present contribution, which is the design, fabrication, and pilot deployment of the low-cost wearable hardware platform.

Second, although YOLOv11n was selected because of its favorable trade-off between detection accuracy and computational efficiency, comprehensive target-device benchmarking on the Raspberry Pi Zero W remains limited. Future work will report frame rate, processing latency, CPU utilization, memory utilization, model format, quantization strategy, and thermal behavior during sustained inference. These measurements are necessary before making strong claims about reliable real-time inference under all clinically relevant operating conditions.

Thermal performance, while adequate during testing, may become a constraint during prolonged operation in high-temperature environments. The current enclosure was fabricated using PLA due to its low cost, ease of fabrication, lightweight properties, and suitability for rapid prototyping. However, PLA may not provide the level of thermal performance, sterilization compatibility, and resistance to hospital-grade disinfectants required for long-term clinical deployment. Future iterations will investigate medical-grade materials that offer improved durability, thermal characteristics, sterilization compatibility, and resistance to commonly used hospital disinfectants while maintaining affordability and ease of fabrication.

Additionally, the current prototype lacks integrated sensors such as inertial measurement units (IMUs) or depth cameras. Incorporating IMUs could provide richer contextual information regarding user movements and workflow transitions, while depth sensing may improve activity recognition and medication workflow understanding. These additional sensing modalities have the potential to enhance contextual awareness and improve the robustness of future medication-error detection systems.

Future iterations of the system will focus on integrating edge AI accelerators (e.g., TPU-based modules), medical-grade enclosure materials, and additional sensing capabilities such as IMUs to enhance contextual awareness and workflow understanding. These improvements will support more sophisticated on-device inference and facilitate deployment in demanding clinical environments. Further validation using annotated datasets will also be conducted to quantify detection accuracy, precision, recall, sensitivity, specificity, false-positive rates, and alarm fatigue, aligning with standard evaluation practices in medical AI systems.

### Implications for low-resource healthcare settings

The deployment at CoRSU Hospital demonstrates that low-cost, context-aware hardware can be effectively integrated into clinical workflows without requiring extensive infrastructure. By leveraging commodity components and open-source design principles, the system provides a scalable pathway toward improving medication safety in resource-constrained environments.

This approach aligns with global health priorities emphasizing affordable, adaptable, and locally maintainable technologies for improving patient safety and healthcare quality [8]. With incremental enhancements, the platform has the potential to evolve into a fully integrated, closed-loop medication safety system while maintaining accessibility for low- and middle-income healthcare settings.

### Conclusion

We have designed, built, and pilot-tested a ∼USD 100 head-mounted camera system that provides real-time feedback on medication-preparation errors in the operating room. Using a Raspberry Pi Zero W, Pi Camera Module v3, PiSugar UPS-battery, and a 3-D-printed enclosure, the device records a first-person view of syringe and vial handling without disrupting clinical workflows—a key requirement for low-resource hospitals.

Eight surgical sessions at CoRSU Rehabilitation Hospital confirmed that the unit captures clear footage of drug labels and hand movements, operates untethered for an entire list, and is comfortable for providers to wear. Clinician feedback suggests the system can reinforce protocol adherence and lower the risk of wrong-drug or wrong-dose events.

While the current prototype is limited by on-board processing power and residual thermal constraints, its open architecture makes future upgrades straightforward: edge-AI accelerators for on-device inference, wireless dashboards for real-time alerts, or direct linkage to electronic health-record systems.

By demonstrating that commodity embedded hardware and additive manufacturing can deliver a context-appropriate safety tool, this study offers a practical pathway for improving medication safety—and, by extension, patient outcomes—in resource-constrained surgical environments.

## CRediT authorship contribution statement

**Solomon Nsumba:** Writing – review & editing, Writing – original draft, Visualization, Validation, Supervision, Software, Resources, Project administration, Methodology, Investigation, Funding acquisition, Formal analysis, Data curation, Conceptualization. **Joan Kaitesi:** Writing – review & editing, Writing – original draft, Validation, Formal analysis, Conceptualization. **Dianah Namutosi:** Writing – review & editing, Writing – original draft, Methodology, Formal analysis. **Pius Kavuma Mugagga:** Writing – review & editing, Methodology, Conceptualization. **Joyce Nakatumba-Nabende:** Writing – review & editing, Supervision, Funding acquisition, Conceptualization. **Peter Nabende:** Writing – review & editing, Supervision, Funding acquisition, Conceptualization.

## Ethics statements

The study was conducted in accordance with the Uganda National Guidelines for Research involving Humans.

### Regulatory clearance.

1. **Mildmay-Uganda Research Ethics Committee (MUREC) — CoRSU field site** REC #**MUREC-2024-378** (valid 30 Apr 2024–30 Apr 2025).
2. **Uganda National Council for Science and Technology (UNCST)** Registration Ref. **SIR360ES** (approval period 10 Jul 2024–10 Jul 2025).

## Funding

This work was supported by the Government of Uganda through the Makerere University Research and Innovations Fund (Mak-RIF).

## Declaration of competing interest

The authors declare that they have no known competing financial interests or personal relationships that could have appeared to influence the work reported in this paper.
